# Does the Aggregation Behavior of Overwintering Paper Wasp Gynes Provide Energetic Benefits?

**DOI:** 10.1007/s10905-025-09895-w

**Published:** 2026-01-13

**Authors:** Helmut Kovac, Astrid B. Amstrup, Helmut Käfer, Anton Stabentheiner

**Affiliations:** 1 Institute of Biology, University of Graz, Graz, Austria; 2 Department of Biology, Aarhus University, Aarhus, Denmark

**Keywords:** Metabolic rate, Activity, Aggregation, Paper wasp gynes, *Polistes*

## Abstract

In temperate regions, paper wasp gynes (*Polistes dominula*) move to protected shelters in late autumn for the purpose of overwintering. Overwintering represents an energetic challenge for insects, which require sufficient energy reserves to survive the cold season. The aggregation behavior exhibited by overwintering insects is associated with a number of benefits, including the buffering of temperature fluctuations and low humidity. This, in turn, has the effect of reducing desiccation stress and improving overwintering success by reducing energetic costs. The metabolic rates of individual insects can vary between separated individuals and aggregations, with a decline in metabolic rate observed within larger groups. We investigated the metabolism of paper wasp gynes in order to detect energy savings during overwintering. We measured the CO_2_ production (measure of energy use) of single wasps and aggregations of wasps at three different temperatures (4, 8, 12 °C). Evidence suggests a close relationship between metabolism and locomotor activity; therefore, activity observations were made during the course of experiments. Metabolic rate increased with ambient temperature in a typical exponential course in both singles and aggregations. However, in contrast to the findings of other studies, no difference in metabolism was observed between singles and aggregations. Furthermore, activity scores revealed higher activity levels in aggregations. This indicates that energetic requirements are not the underlying cause of the aggregation behavior exhibited by overwintering paper wasp gynes.

**Supplementary Information:**

The online version contains supplementary material available at 10.1007/s10905-025-09895-w.

## Introduction

In temperate regions characterized by seasonal changes, certain insects enter a state of quiescence during the winter months. This form of overwintering necessitates the accumulation of a specific amount of energy stores during the autumn months (Hahn and Denlinger 2007; Sinclair 2015; Cohen et al. 2022; Stabentheiner et al. 2024). The insects are dependent on their energy reserves to survive the winter period and the early spring season, when food sources remain scarce. Aggregation behavior is a common phenomenon that is often observed in overwintering insects. In autumn, insects seek sheltered locations in order to protect themselves from extreme weather conditions and predators during the winter months. The benefits of aggregation are multifaceted, including the mitigation of external conditions such as temperature fluctuations and low humidity, as well as the reduction of desiccation stress (Yoder et al. 1992; Yoder and Smith 1997; Brower et al. 2008). Moreover, extant evidence suggests a correlation between insect aggregation during the winter and the survival rates of these insects (Hagen 1962; Brower et al. 2008; Szejner-Sigal and Williams 2022).

The depletion rate of energy stores is determined by metabolic rate, and any factors that increase metabolic costs during winter can lead to increased mortality or decreased reproductive success in the following spring. The metabolic rates of certain insects within aggregations have been observed to decrease with increasing group size, suggesting that aggregating may provide energetic benefits to the insects (Tanaka et al. 1988; Tojo et al. 2005; Su et al. 2007; Waters et al. 2017; Szejner-Sigal and Williams 2022). This phenomenon was demonstrated in overwintering ladybeetles, where aggregation was observed to decrease metabolic rates and conserve winter energy reserves, potentially attributable to a reduction in locomotor activity (Szejner-Sigal and Williams 2022).

The primitively eusocial paper wasps (*Polistes* spp.) are distributed across Europe and have successfully spread worldwide in recent times. A total of more than 200 species of the genus exists across diverse regions across the globe, encompassing both tropical and temperate zones (Carpenter 1996; Pickett et al. 2006). In temperate climates, the wasps exhibit an annual nesting cycle with a social colony period during the summer months and a period of quiescence during the winter. At the end of the colony period, reproductive females (gynes) leave their nests, mate and then move to sheltered places (hibernacles), where they aggregate to overwinter (West-Eberhard 1969). The winter hibernacles serve to protect the wasps from predators, precipitation, and low temperatures. However, they offer only limited protection from extreme ambient temperatures. The period of overwintering is of particular significance for the wasps, which experience a high mortality rate prior to the onset of spring (Rau 1930; Pratte 1982; Starks 2001; Gamboa et al. 2004). It is evident that the provision of nourishment during the autumn period, in addition to the strategic management of resources, assumes a pivotal function in ensuring the survival of organisms during the winter season (Strassmann 1979; Hunt et al. 2003; Kovac et al. 2023; Stabentheiner et al. 2024). Stabentheiner et al. (2024) conducted a comparative study of three *Polistes* species from different climates in order to investigate the energy requirements of hibernation. It was determined that the remaining energy reserves in the spring of the three populations in question appear to be adequate to facilitate resting at the nest or minimal brood care throughout the summer months, though not to support intensive foraging activities. The energy requirements of wasps in their hibernacle are mainly determined by the microclimate. Warmer overwintering temperatures have been demonstrated to result in elevated rates of energy expenditure, which in turn leads to the depletion of stored reserves (Kovac et al. 2023). As demonstrated by Kovac et al. (2023), the cumulative mass-specific energetic costs over the winter were found to be the lowest for a temperate *Polistes* population in comparison with two Mediterranean ones.

The hibernation clusters consist of variable numbers of individuals, ranging from a few to hundreds (Rau 1930, 1931; Pardi 1942; Reeve 1991; Dapporto et al. 2005). Hibernation clusters are typically comprised of wasps from different colonies and, on occasion, even different species (Pardi 1942; Yoshikawa 1963; Starks 2003). In the case of *P. fuscatus* foundresses, preferential association with former nestmates over unrelated females has been observed (Post and Jeanne 1982). In *P. dominula*, it appears that foundresses do not exhibit discrimination against close relatives during the spring season, as evidenced by studies conducted by Pratte (1982) and Röseler (1985). However, as demonstrated by Cervo et al. (2002), some mechanisms to avoid association with unrelated individuals in autumn (before they aggregate in hibernacles) are also present in *P. dominula*. In laboratory experiments, foundresses of this species have been observed to demonstrate a preference for the formation of associative nest foundations with individuals originating from the same locality. The advantages of such aggregations are challenging to evaluate in quantitative terms. However, it is conceivable that such aggregations may enhance active defense mechanisms against predators while also providing a degree of insulation from cold and dry conditions.

The 'resource conservation hypothesis' posits that energetic efficiency is scaled with group size when aggregating (Schoombie et al. 2013). However, this hypothesis has only been the subject in a limited number of studies, with the majority reporting clear advantages of aggregation over individual overwintering (e.g., Szejner-Sigal and Williams 2022). The aim of this study was to compare the metabolic rate of isolated and aggregated paper wasp gynes (*P. dominula*) to assess the potential energy-saving benefits of aggregation behavior during winter. We measured the respiratory metabolism of single separated wasps and grouped wasps in an ecologically relevant temperature range (see Kovac et al. 2023). Additionally, we performed an activity assessment to determine if aggregation behavior lead to a reduction in locomotor activity and thus a decrease in metabolism. The hypothesis tested if aggregation behavior promotes energy savings through reduced metabolic rates and we expected that individuals in aggregations would exhibit lower metabolic rates than isolated individuals. This would indicate energy savings as a potential advantage of this special behavior in overwintering paper wasp gynes.

## Materials and Methods

### Animals

The experiments were conducted with paper wasp gynes (*P. dominula*) in the winter seasons (November and December) from 2022 to 2024. The wasps were collected from their hibernacles (wooden bird boxes) at two different locations in Styria (Austria) a few days prior to the beginning of the experiments. We investigated five different aggregations of wasps and took 16 wasps from each aggregation for one experiment. This process was repeated five times.

### Experimental Procedure

The experiments were conducted with single wasps and with aggregations of 16 individuals (the same wasps used in the "single experiments") at three different temperatures of 4, 8 and 12 °C, which are relevant for overwintering. It was not possible to conduct experiments at temperatures below 4 °C due to technical restrictions of the measurement setup. Measurements were performed only at one test temperature per day. Prior to the beginning of the experiments, the wasps were weighed with an accuracy of 0.1 mg (Shimadzu AUW-120DV, Nishinokyo Kuwabaracho, Nakagyoku, Kyoto, Japan). For experiments conducted with single separated wasps were placed in small plastic tubes (Fig. S1; length 35 mm, diameter 9 mm, volume 2.23 ml) that functioned as measurement chambers. Eight of these measurement chambers were arranged in parallel and placed in a water bath (Julabo F33 HT, JULABO Labortechnik GmbH, Seelbach, Germany) with open lid to allow observation and recording the wasps’ activity. The measurement chambers were submerged in the water for about three hours at a constant temperature with an accuracy of ± 0.1 °C during the experiments. Following submersion in the water bath, the wasps had 60 min for habituation to the test temperature and to calm down. Subsequently, the CO_2_ production of the wasps was measured over a period of 128 min (4 cycles of 32 min). The total duration of a trial was 188 min. The experiments were conducted in the dark between 8:00 and 17:00 (realized by darkening the laboratory). The wasps’ behavior during the experiments was recorded with near-infrared light by means of a night vision video camera (Sony GDR-CX730E, Sony Europe Limited, Vienna, Austria), installed above the water bath, for subsequent evaluation of their activity.

The same individuals were used for both solitary and aggregation trials. Two solitary trials were conducted for each aggregation trial. In any given experiment with singles, a total of eight wasps were measured (eight-channel multiplexer, see below). Subsequent to the initial trial, a second set of eight wasps was measured in an identical manner (8 × 2 = 16). On the first day, the measurements were performed at 12 °C, on the second at 8 °C, and on the third at 4 °C (with the same individuals). Prior to initiation and between experiments, wasps were stored at temperatures ranging from 8 to 10 °C.

After the final measurement of single wasps, the entire cohort of one aggregation (n = 16) was transferred to a larger plastic tube (Fig. S1; length 115 mm, diameter 34 mm, volume 104.4 ml) and left undisturbed until the following day. The measurements were performed similarly for the cohorts, commencing at a temperature of 12 °C on the first day for a period of three hours. The investigation encompassed five replications with 5 distinct aggregations, with each aggregation comprising 16 individuals.

### CO_2_ Measurement

The CO_2_ emission of the wasps was measured during the entire observation period. Carbon dioxide (CO_2_) emission is widely employed as an indirect metric of an organism's metabolic rate. Eight respirometry measurement chambers were connected to an eight-channel multiplexer (RM Gas Flow Multiplexer, Sable Systems, Las Vegas, Nevada, USA), working in a stop-flow measuring arrangement. The multiplexer controlled the sequential flushing and shut-off of the eight metabolic chambers, enabling the concurrent measurement of eight individuals in succession. During the flushing phase, the metabolic chambers were perfused with humidified air (50% rH) at a constant flow rate of 144 ml min^−1^. The duration of the flushing phase of one chamber was 4 min and the closed phase was 28 min (entire cycle 32 min). The multiplexer was connected to a differential infrared carbon dioxide gas analyser (DIRGA; URAS 14, ABB, Zürich, Switzerland) which measured the insects’ CO_2_ release with an accuracy of approximately 2 ppm. To maximize the system sensitivity (i.e., to achieve an accuracy of less than 0.2 ppm), the air flow through the measurement chambers was taken from outside the laboratory. Prior to its entry into the reference tube of the DIRGA, the air was directed through a 10 L container, with the purpose of attenuating variations in CO_2_ content. This was followed by its passage through the pump and mass flow controllers (0–1000 ml min − 1, Brooks 5850 S). Subsequently, the air was transferred to an additional container (5 L), where it underwent further damping of CO_2_ and pressure fluctuations. In order to maintain a relative humidity of about 50% in the measurement chambers, the air was humidified by passing it through two bottles filled with distilled water (for methodical details see Stabentheiner et al. 2012). The air was dried by Peltier-driven cool traps set at 10 °C before entering the URAS reference and measurement tubes, where it was heated to 60 °C. The volumes (nl) of CO_2_ production reported in this paper refer to standard (STPS) conditions (0 °C, 101.32 kPa = 760 Torr). The CO_2_ release was measured and recorded at one-second intervals. At the beginning and at the end of each experimental run, the gas analyser was calibrated automatically in zero and end point using internal calibration cuvettes, and the data were corrected for any remaining drift or offset. For further information pertaining to the measurement of carbon dioxide, please refer to the work of Stabentheiner et al. (2012).

### Data Analysis and Statistics

All calculations and graphics were performed with MS Excel (Microsoft Corporation, Redmond, WA, USA), with Origin 2017 software (OriginLab, OriginLab Corporation, Northampton, MA, USA), and R version 4.2.2 (R Core Team 2024). Curve fittings were performed with the Origin software. The mean CO_2_ values for the evaluated parameters referenced in the findings are derived from curve fittings. The accompanying statistics were generated using Statgraphics software (Statgraphics Centurion XVI, StatPoint Technology Inc., The Plains, VA, USA) and IBM SPSS Statistics (SPSS Inc., Chicago, IL, USA). The quantity of CO_2_ produced was determined by integrating the peaks of CO_2_ release during the flushing phase over time. In order to facilitate a comparison of the CO_2_ production of singles and aggregations, the mass-specific metabolic rate was calculated. The logarithmic CO_2_ values of the two groups were then compared with an analysis of variance (ANOVA) with ambient temperature (T_a_) as the independent variable and metabolic rate as the dependent variable.

The activity of the wasps was assessed for the entire period during which CO_2_ measurements were taken. In the evaluation of the video recordings, the behaviour of the wasps was classified into two categories: "no activity" (resting motionless) or "visible activity" (movement of legs or body parts, grooming or walking). Every action, “activity” or “no activity”, had to go on for a minimum duration of 30 s per minute to be categorised as a discrete behaviour event. In the clusters it was not possible to assess activity of each individual over the entire measurement time because of occasional changes in position. Consequently, four activity categories were established, according to the number of wasps present (A: 0%, B: 1–25%, C: 25–50%, D: > 50% active wasps). The data for single wasps were obtained from the observation of 16 individuals (2 × 8). A comprehensive evaluation of the activity level was conducted in R (R Core Team 2024), employing a model devised through the utilisation of the polr() function from the MASS package:

$$Activity=Temperature+Trial*Group\, category$$

In the context of the experimental design, the “activity” variable was defined as an ordered factorial response variable, with temperature designated as a continuous variable. The trial variable (1–5) and the group category (individual or aggregation) were defined as factorial variables. An analysis of variance (ANOVA) table with a type II analysis of deviance was conducted in order to test the variables of the model. The comparison between singles and aggregations activity was conducted with the Mann–Whitney test. The differences in activity observed as a function of temperature were statistical analysed, employing both the Kruskal–Wallis test and the Bonferroni comparison test.

## Results

**Fig. 1:**
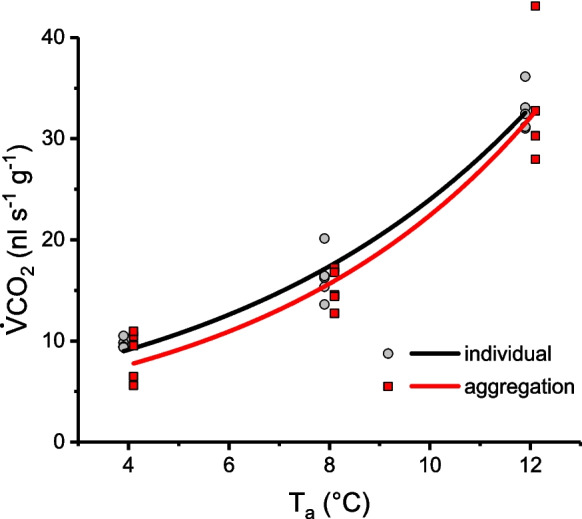
Metabolic rate of paper wasp gynes. Presented are means of CO_2_ production rate (V̇CO_2_ nl s^−1^ g^−1^) of single wasps (n = 16) or aggregations of wasps (n = 16) in relation to experimental ambient temperature (T_a_)

The CO_2_ production increased exponentially with ambient temperature, both in singles and aggregations (Table 1; Fig. 1; Fig. S2). In the single wasps, the mean CO_2_ value (mass-specific, calculated from the fitted curve) increased from 9.0 to 32.6 nl s^−1^ g^−1^, while in the aggregations it increased from 7.8 to 32.8 nl s^−1^ g^−1^ as the temperature rose from 4 to 12 °C. The Q10 values for both were found to be quite similar at 5.8 and 5.7, respectively. After logarithmic transformation of the CO_2_ production values and the application of a comparative ANOVA, we found no statistical difference between the two groups (p >  > 0.05; R-Squared (adjusted for d.f.) = 86.8442%, d.f. = 3; Supporting Information_1 and 2).

**Table 1:** Summary of metabolic rate of paper wasp gynes. Presented are means of CO_2_ production rate (V̇CO_2_ nl s^−1^ g^−1^) of single wasps or aggregations of wasps in relation to experimental ambient temperature (T_a_)

	mean ± SD	V̇CO_2_ (nl s^−1^ g^−1^)
T_a_	individual (N)	aggregation (N)
12	32.8 ± 2.07 (5)	32.9 ± 5.97 (5)
8	16.4 ± 2.39 (5)	15.1 ± 1.86 (5)
4	9.7 ± 0.48 (5)	8.6 ± 2.42 (5)

**Fig. 2:**
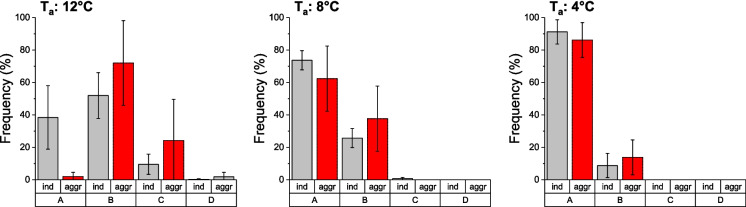
Activity of paper wasp gynes, single wasps (ind, n = 16) or aggregations (aggr, n = 16) of wasps, at three experimental ambient temperatures (T_a_: 12, 8, 4 °C): percentage of active wasps (mean of five trials) during the measurement period (2 h), divided into four categories based on the number of active wasps (A: 0%, B: 1–25%, C: 25–50%, D: > 50%)

The overall statistics showed a significant effect of temperature, trial number, group category, and the interaction between the last two on the activity level of the wasps (Table 2). The activity of the wasps was found to increase with ambient temperature (Fig. 2). At 12 °C the frequency of activity was generally higher than at lower temperatures. Significant differences between temperature treatments were observed for both singles and aggregations in the categories A to C (p < 0.01, Kruskal–Wallis test; Supporting Information_2_Temperature). However, pairwise Bonferroni comparisons showed only differences between 8—12 °C and 4 and 12 °C (p < 0.05, Mann–Whitney test; Supporting Information_2_ Individual vs. Aggregation). The most striking result was that at 12 °C the percentage of the category “no active wasps” (A) was significantly higher in single wasps (38.4%) than in aggregations (2.0%) of wasps (p < 0.01, Mann–Whitney test). In the other test temperatures, no significant differences in percentage of non-active wasps (A) were observed (p > 0.05, Mann–Whitney test). In the other group categories (B – D), there was a general trend that activity was higher in aggregations, but the difference was not significant (Supporting Information_2_Individual vs. Aggregation).

**Table 2:** ANOVA type II statistics of the activity level. Based on a model using “Activity” as an ordered, factorial response variable, “Temperature” as a continuous variable, and “Trial” (1–5) and “Group category” (individual or aggregation) as factorial variables

	*Χ*^*2*^	df	p
Temperature	197.58	4	< 0.001
Trial	257.79	1	< 0.001
Group category	1977.95	1	< 0.001
Trial × Group category	72.65	4	< 0.001

## Discussion

The aggregation behavior exhibited by insects is frequently observed during the winter season, a time when energy conservation is associated with survival (Tojo et al. 2005; Su et al. 2007; Broly et al. 2013; Szejner-Sigal and Williams 2022). The present study contributes to the limited number of studies that have examined the benefits of group overwintering on energy use (e.g., Szejner-Sigal and Williams 2022). In the context of overwintering paper wasp gynes, aggregation has been identified as a general strategy (Rau 1930, 1931; Pardi 1942; West-Eberhard 1969; Reeve 1991; Dapporto et al. 2005). The aggregation observed during winter has been hypothesized to result in substantial energy savings, accompanied by associated fitness benefits.

In the present study the typical exponential increase of metabolic rate with temperature was measured, as previously observed in paper wasp gynes by Kovac et al. (2022). However, in contrast to the results of other studies, no difference was observed between the metabolic rates of single wasps and aggregations (see Fig. 1 and S3). Despite the observed tendency towards reduced metabolic rates in aggregations within trials 4 and 5 (Fig. S2) and significant effects between trial and group category (Table 2), statistical confirmation of the hypothesis that aggregation contributes to energy conservation was not achieved (Table S2). We presume the observed differences in the two trials to be mainly an effect of the year (2024) in which the wasps were collected and not of differently performed experiments.

We had anticipated energetic benefits of aggregation behavior analogous to those observed by Szejner-Sigal et al. (2022) in ladybeetles. Within overwintering aggregations, ladybeetles exhibited a reduced energy expenditure in comparison to single beetles by 57% and 71% with increasing aggregation size ("group effect") at warm and cool temperatures, respectively. The activity of the beetles was found to decrease with aggregation size, but only at low temperatures. The metabolic savings were partly, but not entirely, explained by a reduction in locomotor activity. The reduced energy expenditure observed in overwintering aggregations of the two bugs *Pyrrhocoris apterus* and *Parastrachia japonensis* (Tojo et al. 2005; Su et al. 2007) and the tropical fungus beetle, *Stenotarsus subtilis* (Tanaka et al. 1988), was in similar range due to metabolic savings. Furthermore, the aggregation behavior of woodlice has been observed to have a significant impact on their respiratory rate. Takeda (1984) found that the rate of oxygen consumption per individual decreased with increasing group size, with the rate of consumption being tested for groups of 2, 5 and 10 individuals. Relative humidity within aggregations is a likely mechanism driving metabolic suppression. As posited by Williams et al. (2016), desiccation stress in single beetles may elevate their metabolic demands in comparison to those experienced by beetles inhabiting aggregations. As demonstrated by Tanaka et al. (1988), low relative humidity has been shown to improve metabolic suppression in large aggregations of tropical fungus beetles. These findings suggest that low relative humidity in such aggregations may act as a cue to induce metabolic suppression. However, the extent to which paper wasps are also sensitive to relative humidity while overwintering remains to be elucidated. A study was conducted to ascertain whether field overwintering *Polistes* possess sufficient water reserves (Stabentheiner et al. 2024). This was achieved by measuring the difference between wet and dry mass (an estimation of water loss) in autumn and spring and demonstrated that water reserves are sufficient in early spring (Stabentheiner et al. 2024).

In specific insects, and in the case of overwintering paper wasp gynes, it has been observed that their metabolic rates are reduced prior to entering diapause in order to conserve energy (Irwin and Lee 2003; Hahn and Denlinger 2007, 2011; Sinclair 2015; Kovac et al. 2022). The act of saving energy has the potential to result in augmented energy reserves at the end of winter, a state of affairs that is advantageous to fitness. These energy reserves must allow for the establishment and maintenance of protection against cold, for use in winter metabolism, and for founding a new colony in spring (Stabentheiner et al. 2024). Consequently, the inability to identify a 'group effect' in the wasps, which would concomitantly diminish energy requirements during hibernation, is especially surprising. However, this phenomenon has also been observed in a study of the desert harvester ant *Pogonomyrmex rugosus*, where (mass-specific) oxygen consumption was found to be independent of group size (Lighton and Bartholomew 1988). A comparable outcome was observed in the gregarious caterpillar *Eutricha capensis* (Schoombie et al. 2013), where a comparison was made between singles and groups of individuals. The results indicated that there was no advantage to be gained from aggregation. Furthermore, it was observed that neither the metabolic rate nor the water-loss rate decreased in proportion to an increase in group size. It was observed that the metabolic rate and the rate of water loss remained constant or increased in larger groups relative to singles. Schoombie et al. (2013) posit that other benefits of aggregation (e.g., reduced predation or increased growth rates) are likely to be more significant than energy savings. It is conceivable that this could also be the case for paper wasps. Another reason for aggregation could be that appropriate overwintering places are rare and therefore often used by multiple individuals.

The reduced metabolic rate in aggregating insects may be attributable to reduced locomotion consequent to aggregating behavior. Locomotion is a highly energetic process, which can influence the energetic costs of the entire aggregation. It has been demonstrated in woodlice that aggregation results in a decline in locomotor activity, and consequently, the decrease in metabolic rate may be a consequence of reduced activity (Brockett and Hassall 2005; Dias et al. 2012). The investigation of paper wasp activity in our study yielded contradictory results. In general, the level of activity decreased with decreasing ambient temperature but was still observed at a low level at 4 °C (see Fig. 2). However, in contrast to the findings reported in ladybirds (Szejner-Sigal and Williams 2022) or woodlice (Brockett and Hassall 2005; Dias et al. 2012), we observed higher levels of activity in wasps when they were in aggregations than when they were isolated, particularly at the highest test temperature (Fig. 2). The video recordings demonstrated that in aggregations, the locomotion of one wasp could elicit activity in neighboring wasps, a phenomenon not occurring in isolated wasps. It has been documented that *Polistes* wasps possess sufficient energy reserves in late winter or early spring to sustain the metabolic activity of resting or low-activity individuals (e.g., brood care) for a minimum of one summer season (Stabentheiner et al. 2024). The energetic benefits of grouping are therefore questionable, particularly in the case of *Polistes* aggregations, which do not exhibit the same degree of aggregation as honeybee winter clusters (a phenomenon that is known to conserve energy, Stabentheiner et al. 2003). It is obvious that in the open, unprotected combs of paper wasps even a minimal amount of metabolic heat production is immediately lost to the environment.

In conclusion, our study did not discover any evidence to suggest that aggregation in paper wasp gynes reduces energy requirements. This finding indicates that common biological phenomena, such as aggregation in insects, do not always yield the same benefits across different species.

## Supplementary Information

Below is the link to the electronic supplementary material.

Supplementary file1 (PDF 1.49 MB)

Supplementary file2 (XLSX 18 KB)
