# Supplementary material for: Does the Aggregation Behavior of Overwintering Paper Wasp Gynes Provide Energetic Benefits?
Source: J Insect Behav. 2026 Jan 13;39(1):4. doi: 10.1007/s10905-025-09895-w (PMC12799625; doi:10.1007/s10905-025-09895-w)
Supplement: Supplementary file 1 — Supplementary file1 (PDF 1.49 MB) [file 10905_2025_9895_MOESM1_ESM.pdf]

Does the aggregation behavior of overwintering paper wasp gynes provide energetic benefits?

Helmut Kovac [1], *, Astrid B. Amstrup [1,2], Helmut Käfer [1] and Anton Stabentheiner [1]

[1] Institute of Biology, University of Graz, Austria
[2] Department of Biology, Aarhus University, Aarhus, Denmark
* Correspondence: helmut.kovac@uni-graz.at

Fig. S1 Metabolic measurement chambers for single (left) and aggregations (right) of paper wasp gynes.

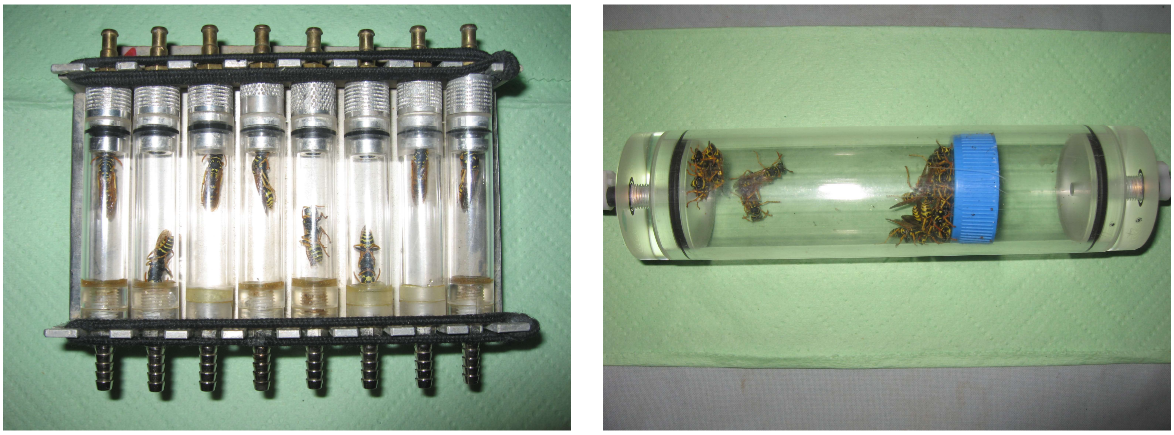

Fig. S2 Metabolic rate of paper wasp gynes. CO_2 production rate ($\dot{V}CO_2$ nl s^{-1} g^{-1}) of single wasps, means of single wasps or aggregations of wasps in relation to experimental ambient temperature (T_a) for all five trials.

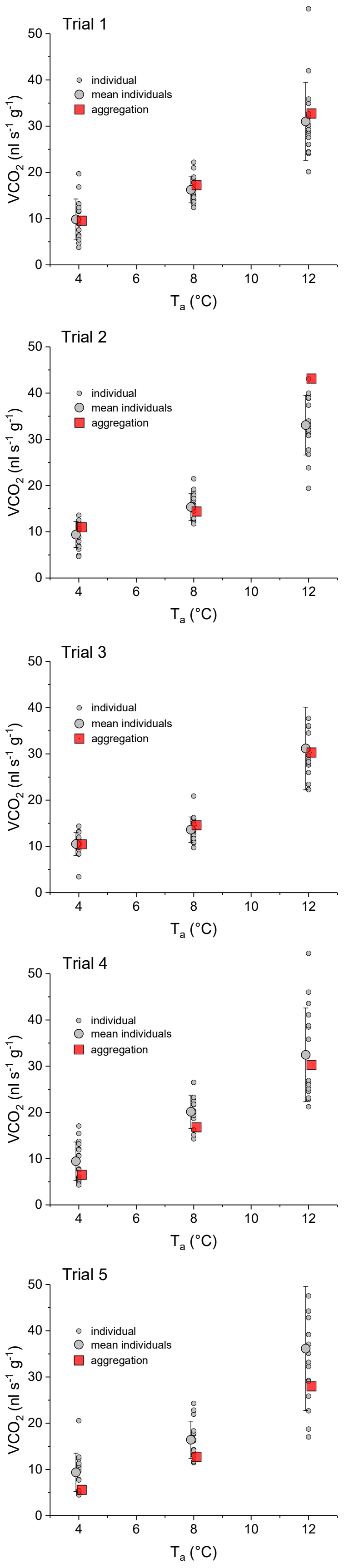

Fig. S3 Metabolic rate of paper wasp gynes. CO_2 production rate (log $\dot{V}CO_2$ nl s^{-1} g^{-1}) of single wasps ($\dot{V}CO_2$ = 0.62636 + 0.06891* T_a) or aggregations ($\dot{V}CO_2$ = 0.61131 + 0.07397* T_a) of wasps in relation to experimental ambient temperature (T_a).

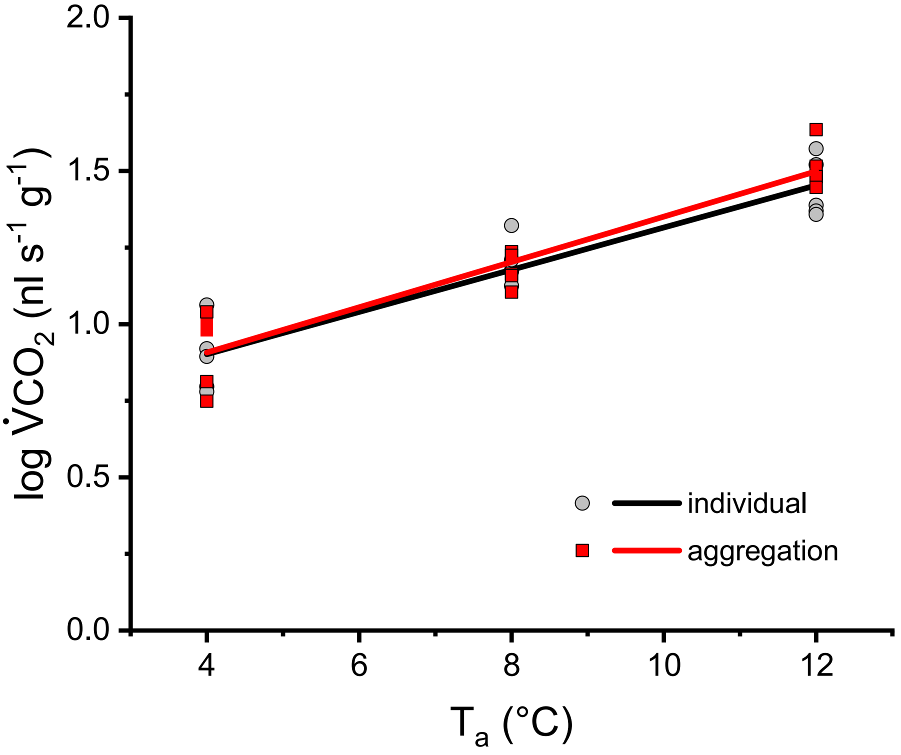

Fig. S4 Activity of single wasps (individual) or individuals in aggregations (aggregation) of paper wasp gynes (*Polistes dominula*) at three experimental ambient temperatures (T_a: 12, 8, 4 °C). Data present the percentage of active wasps during the measurement period (128 minutes), divided into four categories based on the number of active wasps (A: 0%, B: 1-25%, C: 25-50%, D: >50) for all five trials.

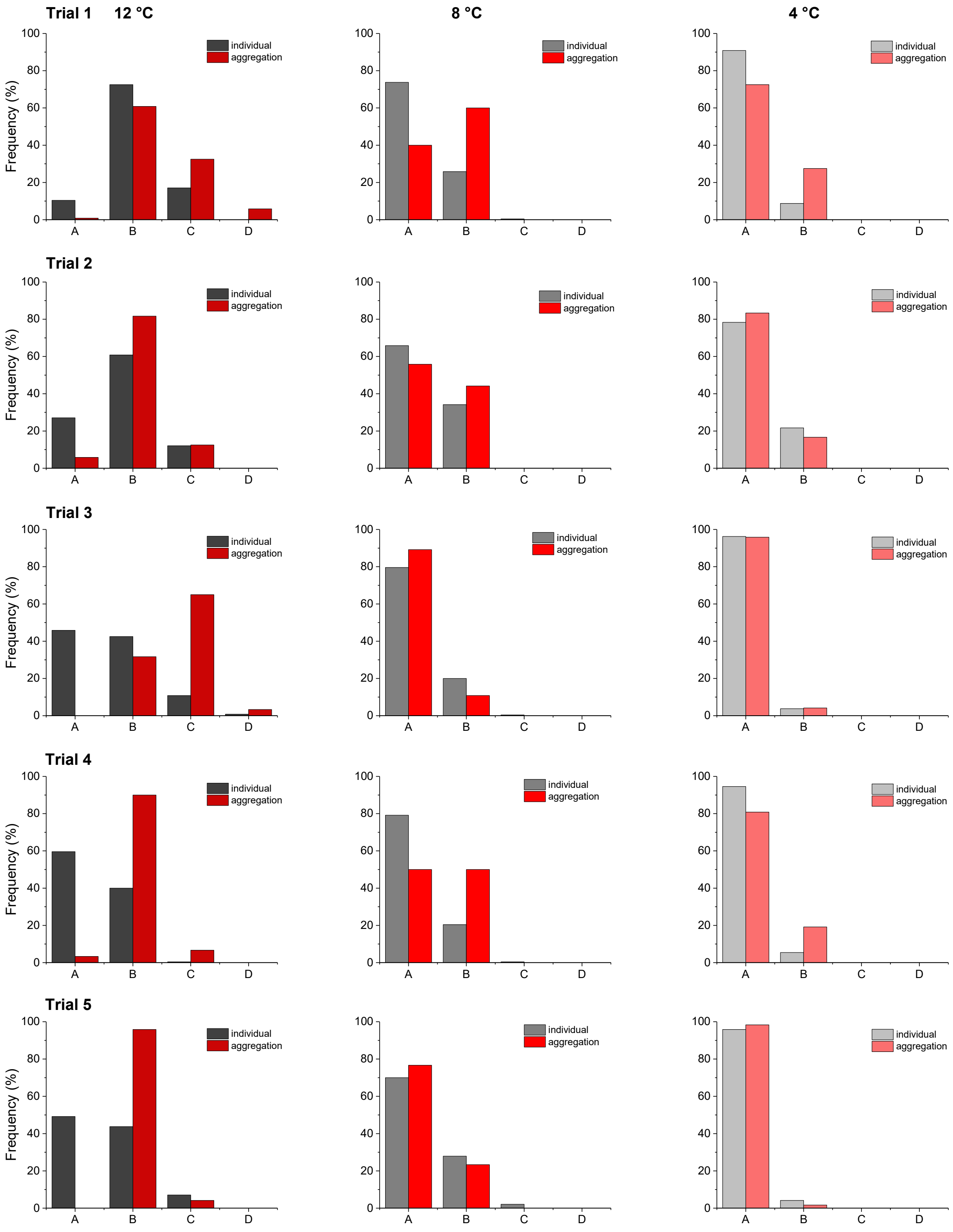

Table S1 Summary statistics of CO_2 production and activity of single wasps (individual) and aggregation of paper wasp gynes.

Date	T_a	Mean CO_2 (nl/s/g)	SD CO_2 (nl/s/g)	Activity_A	Activity_B	Activity_C	Activity_D	Categorie
29.11.2022	12	24.4	8.41	10.4	72.5	17.1	0.0	individual
01.12.2022	8	21.0	2.82	73.8	25.8	0.4	0.0	individual
03.12.2022	4	11.6	4.42	90.8	8.8	0.0	0.0	individual
30.11.2022	12	23.4	8.93	27.1	60.8	12.1	0.0	individual
01.12.2022	8	15.0	2.76	65.8	34.2	0.0	0.0	individual
04.12.2022	4	8.3	2.46	78.3	21.7	0.0	0.0	individual
21.11.2023	12	37.4	6.45	45.8	42.5	10.8	0.8	individual
22.11.2023	8	14.8	2.96	79.6	20.0	0.4	0.0	individual
23.11.2023	4	6.2	2.83	96.3	3.8	0.0	0.0	individual
19.11.2024	12	22.8	10.13	59.6	40.0	0.4	0.0	individual
20.11.2024	8	16.2	3.60	79.2	20.4	0.4	0.0	individual
21.11.2024	4	6.0	4.16	94.6	5.4	0.0	0.0	individual
03.12.2024	12	33.2	13.39	49.2	43.8	7.1	0.0	individual
04.12.2024	8	13.3	4.03	70.0	27.9	2.1	0.0	individual
05.12.2024	4	7.8	4.15	95.8	4.2	0.0	0.0	individual
07.12.2022	12	32.8	2.4	0.8	60.8	32.5	5.8	aggregation
08.12.2022	8	17.2	9.3	40.0	60.0	0.0	0.0	aggregation
09.12.2022	4	9.5	1.5	72.5	27.5	0.0	0.0	aggregation
07.12.2022	12	30.3	7.1	5.8	81.7	12.5	0.0	aggregation
08.12.2022	8	14.6	0.7	55.8	44.2	0.0	0.0	aggregation
09.12.2022	4	10.5	1.9	83.3	16.7	0.0	0.0	aggregation
28.11.2023	12	43.1	1.4	0.0	31.7	65.0	3.3	aggregation
29.11.2023	8	14.4	6.3	89.2	10.8	0.0	0.0	aggregation
30.11.2023	4	11.0	5.5	95.8	4.2	0.0	0.0	aggregation
26.11.2024	12	30.3	2.8	3.3	90.0	6.7	0.0	aggregation
27.11.2024	8	16.8	2.1	50.0	50.0	0.0	0.0	aggregation
28.11.2024	4	6.5	1.6	80.8	19.2	0.0	0.0	aggregation
06.12.2024	12	28.0	5.4	0.0	95.8	4.2	0.0	aggregation
07.12.2024	8	12.7	4.7	76.7	23.3	0.0	0.0	aggregation
08.12.2024	4	5.6	1.0	98.3	1.7	0.0	0.0	aggregation

Table S2 Pairwise comparison of metabolic rate fit curves (Figure S3, log $\dot{V}CO_2$ of nl s^{-1} g^{-1}) of single wasps and aggregation of paper wasp gynes with an ANOVA.

Dependent variable: log CO2< (nl/s/g)

Independent variable: Ta

Level codes: Kat

Number of complete cases: 30

Number of regression lines: 2

Multiple Regression Analysis

Parameter	Estimate	Standard Error	T-Statistic	P-Value
CONSTANT	0.610667	0.0627912	9.72535	0
Ta	0.074	0.00726667	10.1835	0
Kat=individual	0.0146667	0.0888002	0.165165	0.8701
Ta*Kat=individual	-0.005	0.0102766	-0.486541	0.6307

Coefficients

Kat	Intercept	Slope
aggregation	0.610667	0.074
individual	0.625333	0.069

Analysis of Variance

Source	Sum of Squares	Df	Mean Square	F-Ratio	P-Value
Model	1.64273	3	0.547578	64.81	0
Residual	0.219667	26	0.00844872		
Total (Corr.)	1.8624	29			

R-Squared = 88.2052 percent

R-Squared (adjusted for d.f.) = 86.8442 percent

Standard Error of Est. = 0.0919169

Mean absolute error = 0.0710667

Durbin-Watson statistic = 2.00071 (P=0.3018)

Lag 1 residual autocorrelation = -0.0653515

Residual Analysis

	Estimation	Validation
n	30	
MSE	0.00844872	
MAE	0.0710667	
MAPE	6.533	
ME	-1.07322E-16	
MPE	-0.675988	

Further ANOVA for Variables in the Order Fitted

Source	Sum of Squares	Df	Mean Square	F-Ratio	P-Value
Ta	1.63592	1	1.63592	193.63	0
Intercepts	0.00481333	1	0.00481333	0.57	0.4572
Slopes	0.002	1	0.002	0.24	0.6307
Model	1.64273	3			
